# Machine learning classification of plant genotypes grown under different light conditions through the integration of multi-scale time-series data

**DOI:** 10.1016/j.csbj.2023.05.005

**Published:** 2023-05-23

**Authors:** Nazmus Sakeef, Sabine Scandola, Curtis Kennedy, Christina Lummer, Jiameng Chang, R. Glen Uhrig, Guohui Lin

**Affiliations:** a Department of Computing Science, University of Alberta, Edmonton, Alberta, Canada; b Department of Biological Sciences, University of Alberta, Edmonton, Alberta, Canada; c Department of Biochemistry, University of Alberta, Edmonton, Alberta, Canada

**Keywords:** Phenotyping, Computer vision, Machine learning, Deep learning, Feature extraction

## Abstract

In order to mitigate the effects of a changing climate, agriculture requires more effective evaluation, selection, and production of crop cultivars in order to accelerate genotype-to-phenotype connections and the selection of beneficial traits. Critically, plant growth and development are highly dependent on sunlight, with light energy providing plants with the energy required to photosynthesize as well as a means to directly intersect with the environment in order to develop. In plant analyses, machine learning and deep learning techniques have a proven ability to learn plant growth patterns, including detection of disease, plant stress, and growth using a variety of image data. To date, however, studies have not assessed machine learning and deep learning algorithms for their ability to differentiate a large cohort of genotypes grown under several growth conditions using time-series data automatically acquired across multiple scales (daily and developmentally). Here, we extensively evaluate a wide range of machine learning and deep learning algorithms for their ability to differentiate 17 well-characterized photoreceptor deficient genotypes differing in their light detection capabilities grown under several different light conditions. Using algorithm performance measurements of precision, recall, F1-Score, and accuracy, we find that Suport Vector Machine (SVM) maintains the greatest classification accuracy, while a combined ConvLSTM2D deep learning model produces the best genotype classification results across the different growth conditions. Our successful integration of time-series growth data across multiple scales, genotypes and growth conditions sets a new foundational baseline from which more complex plant science traits can be assessed for genotype-to-phenotype connections.

## Graphical Abstract

**Figure:**
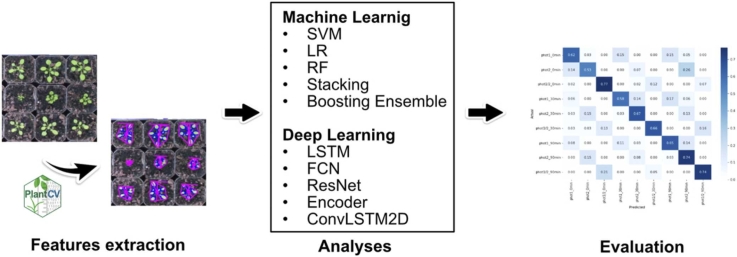

## 1 Introduction

Evaluation, selection, and production of crop cultivars all heavily rely on genotype classification [1]. To meet the ever-increasing global demands for food, plant productivity must be drastically improved by using resources more effectively. However, to do this requires a thorough grasp of genotype-phenotype connections, which relies on effective phenotyping [2]. Phenotyping is critical for the identification of diseased plants, classification of different species or cultivars/genotypes, and/or measurement of plant traits resulting from the interaction of a plant’s genotypes with the environment [3], [4], [5]. Identification of plant genotypes based on phenotypic data is a necessary step in plant variety selection and plant stress analysis, however, when performed manually can be prone to errors and bias. To overcome this, numerous image processing, computer vision, traditional machine learning (ML), and deep learning (DL)-based algorithms can be utilized for plant trait estimation and classification tasks in order to remove human interaction and to increase overall accuracy [4].

In plant classification studies, time-series data is particularly valuable, as it provides important temporal information such as plant productivity over a season and a plant’s dynamic growth based on leaf area/perimeter [6], [7], [8]. Correspondingly, the incorporation of time-series classification is crucial for effective computer vision and machine learning implementation in generating genotype-phenotype connections [9], [10], [11], [12]. Plant phenotyping has predominantly utilized traditional image processing and modeling techniques; however, more modern machine learning-based systems have demonstrated improved accuracy. For example, studies demonstrating the utility of computer vision have looked at plant classification based on whole plant image processing, vesselness measurements, images of overlapping leaves, as well as plant texture features [10], [11], [12], [13], [14]. Furthermore, research applying computer vision methods has succeeded in identifying plant diseases based on probabilistic classification of “healthy” vs “sick” images of plants with diseased areas, signs of environmental stress, and by identifying changes in the electrical signals of plants due to environmental changes [15], [16], [17], [18], [19].

In plant analyses, computer vision and ML techniques have proven their capacity to learn plant growth patterns [4], [5]. For example, Random Forest (RF) ML algorithms have been applied to disease prediction, protein sequence selection, and gene selection as well as plant biomass prediction using image-based data [20], [21], while Support Vector Machine (SVM) has also been applied to identify plant stress based on image data, neuro-image classification, plant image classification, and biomass prediction [18], [22], [23]. As well, stacking multiple ML classifiers has demonstrated additional advantages for crop categorization estimation when compared to the use of a single classifier, suggesting that multiple classifiers in combination can lead to more robust classification outcomes [24], [25].

The plant sciences have also steadily incorporated DL methods, with experimentation using DL algorithms providing superior performance relative to conventional ML algorithms in classifying plants and in detecting various plant diseases [26], [27], [28]. Convolutional Neural Networks (CNNs) have successfully classified plants [29], [30], [31], [32], [33] and identified diseased plants [34], [35], [36]. For example, a CNN-based approach DenseNet-77 gave better accuracy than SVM and K-Nearest Neighbors (KNN) in detecting diseased plants [35]. CNN techniques have also been proven capable of differentiating plants according to species [27], [31], [32], [33], [37]. Recurrent Neural Networks (RNN) have also been successful in analyzing spatiotemporal data when paired with CNNs [38], [39], [40]. Long Short-Term Memory (LSTM; an RNN variation), has also been used for sequential data tasks due to its ability to capture long time-frame dependencies [41], while LSTM and Bidirectional-LSTM (the improved architecture of LSTM) approaches have successfully aided in assessing rice cultivation in southern Brazil [42]. Here, the authors compared their results with classic ML methods, including SVM, RF, k-Nearest Neighbors (k-NN), and Normal Bayes (NB). Based on the Densenet201 and bi-directional LSTM, a Densenet201-BLSTM model was proposed for classifying various genotypes based on time-series of plant images [1]. In the model plant *Arabidopsis thaliana*, a CNN-LSTM method proved most useful in classifying four accessions (Sf-2, Cvi, Landsberg (Ler-1), and Columbia (Col-0)) for plant growth differences and to categorize genotypes over plant development using single images over multiple days [3]. Alternatively, CNN with convolutional-LSTM (ConvLSTM) layers also demonstrated success when re-analyzing the same data [4].

*Arabidopsis thaliana* is a model plant species with extensive, well-characterized genetic resources for use in training ML and DL models [3], [4]. Critically, plant growth and development are highly dependent on sunlight, rendering our ability to detect genotype-to-phenotype differences fundamentally connected to light detection and core to agricultural applications. Light energy provides plants with a means to photosynthesize for growth [43] and a means to detect the environment for development [44]. Plants detect light signals, such as changes in light amplitude, color, spectra, and photoperiod, using a class of proteins called photoreceptors [45]. This enables plants to respond to changes in their environment, such as seasonal transitions, day-night cycles [46], or shade from other plants [47]. There are four families of photoreceptors: Phytochromes (PHYA - PHYE), Cryptochromes (CRY1, CRY2, and CRY3), Phototropins (PHOT1 and PHOT2), the ZTL/FKF1/LKP2 group proteins, and lastly the UV-B resistance 8 (UVR8) family proteins [45], [48], [49]. Phytochromes absorb light in the red and far-red regions of the visible spectrum [50], [51] and regulate key developmental events such as seed germination, timing of flowering, plant size and shape as well as leaf movement [52], [53], [54], [55], [56], [57], [58], [59]. Cryptochromes detect blue and UV-A light [60]. They function during de-etiolation (the transition to the greening stage after plant germination; CRY1), in the photoperiodic control of flowering (CRY2), in the inhibition of the hypocotyl growth and in shade avoidance mechanism (CRY1 and CRY2) [61], [62], [63], [64]. Phototropins and ZTL/FKF1/LKP2 group proteins are sensitive to blue light. Phototropins control phototropism, light-induced stomatal opening, and chloroplast movement in response to changes in light intensity and direction [65]. Lastly, the ZTL/FKF1/LKP2 group proteins promote degradation or maintenance of circadian transcription factors, induce transitions in the day-to-night transition [66], and are also involved in flowering, while the UVR8 family proteins absorb UV-B to signal harmful ultraviolet radiation [67]. While much has been resolved about how light activates plant photoreceptors, there are still gaps in our understanding of how these photoreceptors are connected to different elements of diel plant cell processes to affect phenotypic changes [68].

As plants are not static, but constantly growing and developing on a daily basis, our study aims to make genotype-to-phenotype connections using time-series growth data across multiple scales (intra- and inter-day data), genotypes, and growth conditions. To do this, we grew 17 different photoreceptor mutants under different light conditions to define the effects of twilight on a well-characterized population of photoreceptor deficient plants. To categorize these 17 genotypes based on their phenotypic responses under these different twilight conditions, we extensively tested a number of ML models, including: Support Vector Machine (SVM), Logistic Regression (LR), ensemble stacking techniques (Random Forest (RF) and Boosting), alongside multiple DL techniques such as Fully Convolutional Network (FCN), Resnet, Encoder, Bi-LSTM, Conv2D, and ConvLSTM2D. We find that although conventional ML models were successful in categorizing genotypes under different twilight lengths, DL techniques perform much better, which we attribute to their ability to utilize multiple types of time-series data. The optimal parameter settings for each of the conventional and deep learning models we provided for classifying plants were identified. To make the results easier to grasp, we cross-validated the results and displayed them in boxplots. In particular, our results demonstrate that while SVM maintains greater accuracy in classification tasks, the combined ConvLSTM2D model produced the best classification results for the various genotype classes of *Arabidopsis thaliana* across the different twilight conditions.

## 2 Materials and methods

### 2.1 Plant material and growth conditions

**Fig. 1:**
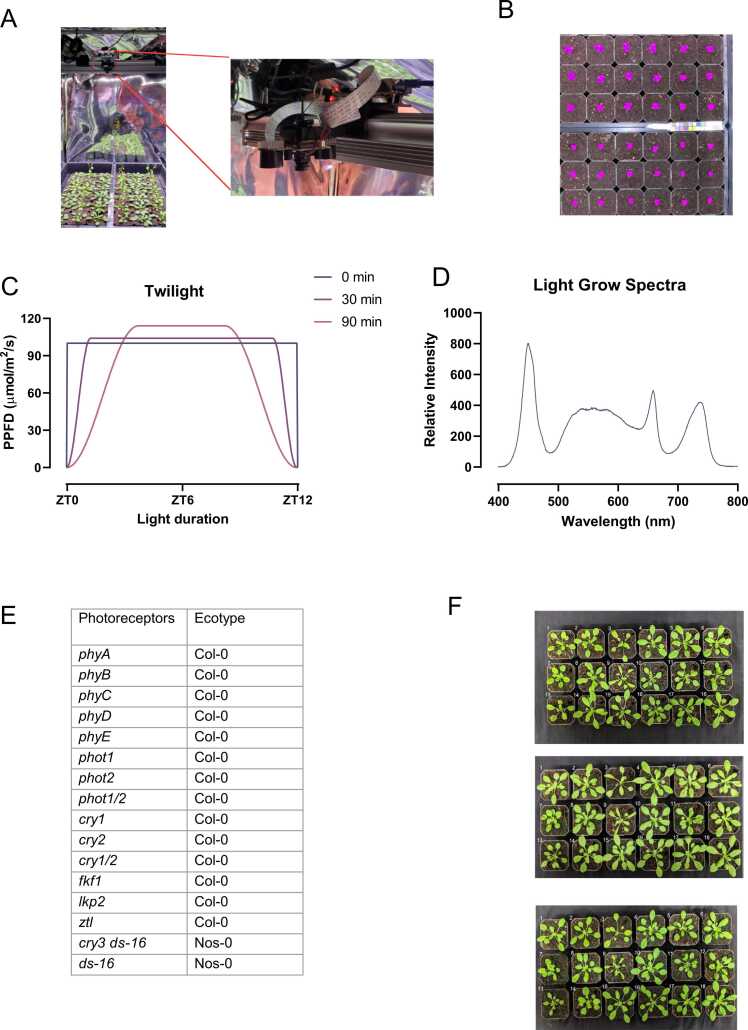
**:** Lights treatments and plant phenotype. (A) Example depiction of the experimental light and camera setup. B) Example depiction of PlantCV data extraction. C) Twilights employed in the study. For the 0 min square bracket ramp, lights turned on to full Photosynthetic Photon flux Density (PPFD) or light intensity, while under the 30 min and 90 min conditions, the light intensity progressively increased to reach its maximum in 30 min and 90 min respectively. For all conditions, the plants received the exact amount of light of 4.32 DLI (mol/m^2^/d). (D) Spectral composition of the light for all treatments. (E) Plant list and associated genotypes. (F) Pictures of representative photoreceptor deficient plants grown under different twilight lengths. Plant position: 1 = WT, 2 = *phyA*, 3 = *phyB*, 4 = *phyC*, 5 = *phyD*, 6 = *phyE*, 7 = *phot1*, 8 = *phot2*, 9 = *phot1/2*, 10 = WT, 11 = *cry1*, 12 = *cry2*, 13 = *cry1/2*, 14 = *cry3 ds-16,* 15 = *ds-16*, 16 = *fkf1*, 17 = *lkp2*, and 18 = *ztl*.

Experiments were performed using *Arabidopsis thaliana* plants, ecotypes Columbia (Col-0) and Nossen (No-0). Photoreceptor mutants (*phy A*, *phy B*, *phy C*, *phy D*, *phy E*, *phot 1*, *phot 2*, *phot1 phot2*, *cry1*, *cry2*, *cry1 cry2*, *cry3*, *ds-16*, *fkf1*, *lkp2,* and *ztl*) (Fig. 1) were obtained from Dr. Enrico Scarpella (University of Alberta, Edmonton, Alberta). Seeds were sterilized in an air-tight container filled with chlorine gas for 24 h. Chlorine gas was made by adding 3 mL of hydrochloric acid to 75 mL of bleach. The seeds were then placed on a ½ Murashige and Skoog media containing 7 g/L of agar (Phytoblend, Caisson) and 1% sucrose at pH 5.8 (KOH). After 3 days of stratification at 4 °C in the dark, the seeds were exposed to light treatment for a week before being transferred to soil (Sungro, Sunshine Mix® #1). Growth chambers were equipped with a programmable Perihelion LED fixture (G2V Optics Inc.) and lined with Reflectix® to ensure a good light diffusion. Plants were grown under a 12 h light and 12 h dark photoperiod with a temperature of 21 °C during the day and 19 °C at night. Light treatments consist of different dawn and dusk ramp conditions for a given spectrum (Fig. 1 A and B). Six LED types (Cree LED XPE, XPE2 XPG families) were used in the fixtures, with characteristic wavelengths of 444 nm, 630 nm, 663 nm, 737 nm, 3000 K white, and 6500 K white.

### 2.2 Phenomics measurements

Each chamber was equipped with two Raspberry Pi 3 B+ and an ArduCam Noir Camera (OV5647 1080p) with a motorized IR-CUT filter and two infrared LEDs. Pictures were taken every 5 min over 14 d and were used to extract plant area measurement using PlantCV (https://plantcv.readthedocs.io/), an open-source Python package with computer vision tools for plant phenotyping as previously described [69].

### 2.3 Dataset construction

To create our dataset, images were processed using PlantCV [69]. The workflow consists of three core steps. First, image preprocessing was required to undistort the fisheye effect of the ArduCam Noir Camera. Images also received exposure correction using a defined white spot region of interest (ROI). The second core step was image segmentation, in which pixels were classified into ‘plant’ and ‘background’ using an HSV color threshold. Segmented images were then filtered to remove small noises. The final core component of our PlantCV workflow was image analysis. Using the segmented images, measurements were taken of the plant dimensions and stored in an output CSV file. The measurement of a size marker was also taken, which served as a constant. This allowed us to normalize the data across different cameras and account for slight variations in the distance between the plants and the camera. Additionally, if the size marker has a known size, it allows the measurements to be converted from pixels to SI units.

### 2.4 Input data structure

Our dataset utilizes 17 genotypes (501 total plants) imaged every 5 min over 14 days of growth. In a DL time-series classification model, input data arrays from the input nodes are transmitted to the hidden layer of the network model, where the network model processes and learns the historical pattern before producing an accurate prediction. The time-series of each chosen station variable was normalized to a similar range of values to guarantee that the proposed sequential neural model is appropriately trained and converges quickly. Additionally, the input data arrays at various sample rates produced negative impact on the model training and validation performances since the collected time-series observations were noisy and stochastic in nature. In our plant dataset, we found inherent biological variance in plant sizes with respect to area. To increase the model’s capacity for generalization, we used data normalization technique [70]. Here, we used the min-max normalization technique [71], where the initial data was transformed linearly using min-max normalization, also known as feature scaling. Using this method, we obtained all data scaled within the range (0, 1). That is, every feature’s minimum and maximum values were each converted to a 0 and a 1, respectively, while all other values were converted to a decimal between 0 and 1, using the formula:

*X* = (*Xi* – *Xmin*) / (*X* *max* – *X* *min* )

where *Xi* is the area value (>0) at a given time for a particular plant; *X*min and *X*max are the minimum and maximum area values per plant respectively; *X* is the normalized plant datasets.

### 2.5 Proposed methodology

We proposed various machine learning models for our research. We separated them into conventional machine learning models and deep learning models to classify different genotypes of A. thaliana, and we tested their performance throughout different dawn and dusk periods of the plants (Figure 2.2). We used hyperparameter tuning to find out the optimal settings of each model to generate the best classification accuracy. Moreover, we cross-validated all the results using 10-fold cross validation techniques. Finally, we evaluated our models using precision, recall, f1-score, and accuracy metrics.

### 2.6 Traditional machine learning (ML) models

#### 2.6.1 Support vector machine (SVM)

SVM models work by finding the best separating (maximal margin) hyperplanes between the classes of training samples in the feature space. SVM accepts input a set of data points in a vector space. In our case, a data point.

represents the plant area at a particular time point, and each dimension represents the total growth feature of the plant. Our proposed SVM model requires 4 parameters to be determined to provide accurate class predictions. While training with SVM model, the proper kernel function for maximizing the margin, regularization parameter (C) as decision function at classifying points, and gamma parameter for defining influence of a single training sample should be considered. To do this, we used a grid search method to find the optimal parameter setting of SVM for achieving the maximum accuracy with the minimum error. Ranges were set to [1], [10] at increment of 1.0 for C and [0.01, 0.001, 0.0001, 0.1–0.5] for ε with γ being fixed at 0.5. The optimal values of C, ε, and γ are selected using 10-fold cross-validation repeated ten times to increase the reliability of the results. We tried SVM with several different kernels for our experiment and found that polynomial kernels with degree 4, C= 10, ε = 0.1, and γ = 0.5 delivered the best precision, recall, accuracy, and F1-score values. Proposed SVM model works by mapping the time-series plant area data to a higher-dimensional feature space using a polynomial kernel function which allows the model to capture non-linear relationships between the time-series plant area data and the genotype labels. As this is a multiclass classification problem, SVM finds multiple hyperplanes that collectively best separate the data points into the designated number of classes. We set the “multi class” argument to “ovr” for the one-vs-rest strategy. In our experiment, the SVM model employed the normalized area values of plants for training and testing as described above.

#### 2.6.2 Logistic regression (LR)

LR works by estimating the probabilities of events, including determining a relationship between features and the probabilities of outcomes. Here, we used multinomial logistic regression to accommodate multi-genotype classification problems. Both the training and testing phases of the logistic regression (LR) model employed the normalized area values of plants for training and testing as described above. Here, for out study, the input to the LR is a total plant as independent variable and the dependent variable is the 17 genotype classes. The input plant area values with randomly initialized weight values added with bias term is passed to sigmoid function and mapped to a probability in a range between 0 and 1. Multinomial logistic regression alters the logistic regression model to directly support the prediction of multiple class labels. To more specifically forecast the likelihood that a given input example (i.e., a particular plant) falls under each accepted class label (*wt, phyA, phyB*, etc.) and the class label with highest likelihood value is selected. We split the dataset into 10-fold for training and validating the model's performance and finally, we reported the maximum accuracy with the minimum cross-validation error. The performance of LR model greatly depends on the hyper-parameter C, the number of iterations, penalty, and the solver. Ranges were set to [0,4] at increment of 0.2 for C and *[‘l1′, ‘l2′*] for the regularization parameter, *[‘newton-cg’, ‘lbfgs’*] as classifier solver. By grid search, we found the best parameter setting for logistic regression with *C = 0.62, max_iteration = 200,* and *solver = “lbfgs”* with *l*_*2*_ regularizer. As this is a multiclass problem, we set the parameter *“multi_class = multinomial”*; the solver *(“lbfgs”)* handled multinomial loss, and 200 iterations were taken for the solver to fast converge.

#### 2.6.3 Random forest (RF)

Random Forest (RF) is an ensemble classification algorithm that consists of a group of tree-based classifiers *h*(*x,* (*θ*)_*k*_), k = 1, 2,., where x is the input vector and (*θ*)_*k*_ are independent and identically distributed random vectors [72]. The decision trees are built using the selected features and training data. At each node of the tree, the best split is selected based on Gini impurity for our proposed model. Where Gini impurity denotes importance or feature relevance, is used to depict the results of feature selection. During testing, each decision tree makes its own prediction, and the final prediction is the one that is most commonly predicted across all the trees. The algorithm achieves higher performance on high dimensional data by doing an implicit feature selection using a small collection of “strong variables”. Random Forest (RF) highly depends on *“max_features”, “n_estimators”, “min_sample_leaf”, and “oob_score”* parameters to provide accurate classification performance [73]. Here, “*n_estimators*” denotes the number of trees the algorithm builds before averaging the predictions, “*max _features*” denotes the maximum number of features random forest considers splitting a node, *min _sample_leaf* determines the minimum number of leaves required to split an internal node, and “*oob_score*” is a random forest cross-validation method. Here, we adjusted these parameters to run the RF model for our analyses, optimized by grid search parameter tuning. The primary parameters are the number of predictors at each decision tree node split and the number of decision trees to run. We used “max*_feature = Auto”* to consider all the data points in individual runs, *“n_estimators = 200″* for better classification performance as well as faster running time, and “*oob_score = TRUE*” to use the RF cross-validation method. After trying multiple leaf sizes, we chose *“min_sample_leaf = 20″* to achieve maximum accuracy with minimum error for validation data for Random Forest (RF) model. RF model employed the normalized area values of plants for training and testing.

#### 2.6.4 Boosting ensemble

ML models can be used individually or as part of an ensemble to fit data. A new model with greater effectiveness is created by the ensemble of simple individual models. ML boosting can create an ensemble [74]. Boosting gives a prediction model in the form of weak prediction models, which are typically decision trees (DT). By learning straightforward decision rules derived from previous data, decision tree builds training model that may be used to predict the class or value of the target variable (training data). To predict a class label, decision trees begin at the tree’s root and compare the root attribute’s values with that of the attribute on the record. Then it follows the branch that corresponds to that value and goes on to the next node based on the comparison. Boosting creates an ensemble model by gradually integrating a number of weak decision trees. It gives each tree’s output a weighted rating. Then, it increases the weight and input for inaccurate classifications from the initial decision tree. The boosting technique combines these several weak prediction rules into a single strong prediction rule after many cycles. For our study, we used Gradient Boosting (GB) technique. By creating base learners (decision trees) consecutively, GB improves the loss function so that each base learner is always more effective than the preceding one. GB method produces accurate results at the beginning rather than fixing mistakes as they occur. Because of this, GB results in more precise results. For grid search, ranges were set to [10,500] with an increment of 10 for “*n_estimators*”, [0.0001, 0.001, 0.01, 0.1, 1.0] for “*learning_rate*”, [0.5, 0.7, 1.0] for “*subsample*”, and [3], [7], [9] for “*max _depth*” parameter. Finally, we used “*n_estimators = 200*” denotes the number of decision trees that are added to the model sequentially to correct and improve upon the predictions made by prior trees, “*max _feature = Auto*” determines the maximum number of features decision trees considers splitting a node, for our case, we are considering all the features in a single run. We set “*learning_rate= 0.01*” to update model weight and biases for convergence, “*loss=log_loss*” for better classification performance, “*max _depth = 7*” denoting max number of levels in each decision tree, and “*subsample = 0.5*”. Boosting ensemble employed the normalized area values of plants for training and testing.

#### 2.6.5 Stacking

In stacking, predictions from various machine learning models are combined on the same dataset. The architecture of a stacking model consists of two or more base models [75], frequently referred to as level-0 models (in our study, they are RF, LR, and Boosting), and a meta-model, commonly known as level-1 model (which is SVM with polynomial kernels in our study), that combines the predictions of the base models. The outputs from the base models that are utilized as input to the meta-model are probabilistic values or, in the case of classification, class labels (i.e., genotypes). The meta-model takes these input features and makes the final prediction for the plant genotype. We prepared the training dataset for the meta-model via 10-fold cross-validation of the base models, where the out-of-fold predictions are used as the basis for the training dataset for the meta-model.

### 2.7 Deep learning models

#### 2.7.1 Fully convolutional network (FCN)

We employed the architecture suggested by Wang et al. [10], which is comprised of three convolutional blocks, each of which has three operations (a convolution, a batch normalization, and then a ReLU activation function [76]). Over the complete time-series, which corresponds to the Global Average Pooling (GAP) layer, the third convolutional block's output is averaged. The output of the GAP layer is then fully coupled to a conventional *softmax* classifier with the same number of neurons as classes in the dataset [77]. The exact length of the time-series after the convolution is preserved by all convolutions having a stride of 1 and zero padding. The first convolution has 128 filters with filter lengths of 8 and 256 filters with filter lengths of 5, respectively. These two convolutions are then input to the third and final convolutional layer, which has 128 filters with filter lengths of 3.

#### 2.7.2 Residual network (ResNet)

**Fig. 2:**
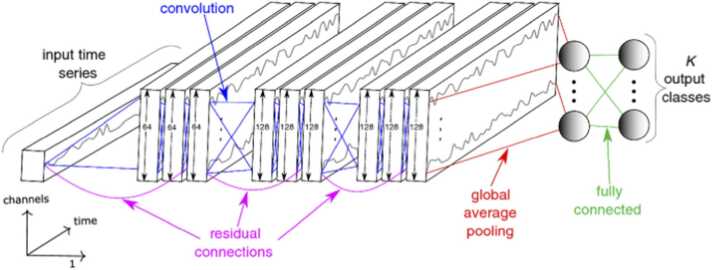
**:** The Residual Network’s architecture for time-series genotype classification of plants.

The deepest architecture for time-series classification is ResNet [10], which has 11 layers, the first 9 of which are convolutional and are followed by a Global Average Pooling (GAP) layer that averages the time-series over the time axis, minimizing the influence of vanishing gradients [78]. Our ResNet architecture consisted of three residual blocks, a GAP layer, and a *softmax* classifier with the same number of neurons as the number of genotype classes in the dataset as its final component. Each residual block is made up of three convolutions, the output of which is added to the input of the residual block and fed to the following layer. With the ReLU activation function being preceded by a batch normalization procedure, the total number of filters for all convolutions is fixed at 64. The length of the filter in each residual block is set to 8, 5, and 3 for the first, second, and third convolutions, respectively. Fig. 2 represents the ResNet architecture that we used in our study of genotype classification.

#### 2.7.3 Long short-term memory (LSTM)

LSTM architecture provides outcomes for sequential data workloads thanks to its capacity to capture long-time dependencies [41], [42]. Here, we used the LSTM and Bi-LSTM (improved architecture of LSTM) with two hidden layers (256 LSTM units) and *relu* activation function for input and dense layers. For the output layer, we used *softmax* activation function having the same number of neurons as the number of genotype classes in the dataset. We trained the models with the following hyperparameters: (a) 250 epochs (after that no changes in validation loss or validation accuracy); (b) dropout rate of 0.3; (c) Adam optimizer [79] with a starting learning rate of 0.01; and (d) mini-batch size of 32. Additionally, categorical cross-entropy loss function was applied to monitor the validation loss.

#### 2.7.4 Encoder

**Fig. 3:**
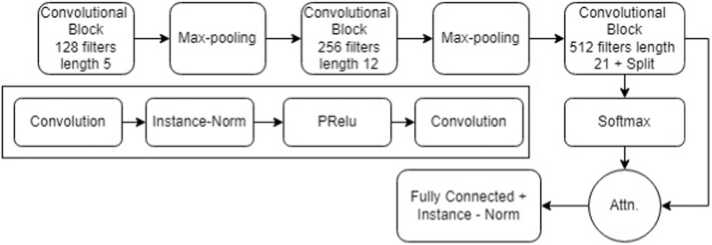
**:** Encoder architecture for time-series genotype classification of plants.

Our proposed encoder model is a standard convolutional network, with a convolution attention mechanism to summarize the time axis. We employed an encoder model with the following architecture (Fig. 3). Our model has 3 convolution layers. The first layer is made up of 128 filters with a length of 5, the second one is made up of 256 filters with a length of 11, and the third one is made up of 512 filters with a length of 21. We used the Parametric Rectified Linear Unit (PReLU) activation function [80] that feeds the output of each layer’s instance normalization operation. A dropout operation (with a rate of 0.2) and a final MaxPooling of length 2 are performed after PReLU’s output. An attention mechanism that allows the network to learn which time-series (in the time domain) are essential for a specific classification was fed the third convolutional layer (Fig. 3). Half of the 512 filters of last convolution layer are input to the timewise *softmax* activation, which acts as an attention mechanism for the other half of the filters. Encoder represents a hybrid deep CNN [81], which is distinguished from FCN by replacing the GAP layer with an attention layer, minor modifications in convolutional layers, the PReLU activation function, the dropout regularization method, and the max pooling procedure. We applied 10-fold cross-validation on the datasets and validated the performance of encoder model by monitoring categorical cross-entropy loss of the validation set.

#### 2.7.5 Conv2D

For long non-linear input sequences, Conv2D model performs well. The 2-D MaxPooling layer and 2-D kernel size are features of the Conv2D model [70]. The Conv2D input shape for the input layer was obtained using an *input shape* argument below:

(1) Input Shape Conv2D = (batch, timesteps, features)

We constructed a 7-layer Conv2D model to consist of an input layer, two 2-D MaxPooling layers, one convolution hidden layer, one flatten layer, one dense layer, and finally output the layer (2nd connected dense layer). The MaxPooling layer reduced the spatial dimensionality of the input sequence volume through down-sampling approach. The flatten layer used the processed input sequence from the previous layer to narrow the featured sequence by wrapping it as a 1-D vector. Our Conv2D model had 128, 64, and 72 convolutional filters (layer neurons) for the input, hidden, and dense layers respectively, using a dropout of 0.3. The input, hidden, and dense layers were activated using the *relu* function, whereas the output layer was activated using the *softmax* function having the number of neurons equal to the number of genotypes in the dataset.

#### 2.7.6 ConvLSTM2D

For the ConvLSTM2D model, the input data should maintain the following shape [70]:

(2) Input Data ConvLSTM2D = X·reshape(samples, Input Shape ConvLSTM2D) = *X·reshape*(*samples, batch, timesteps, features, channels*)

**Fig. 4:**
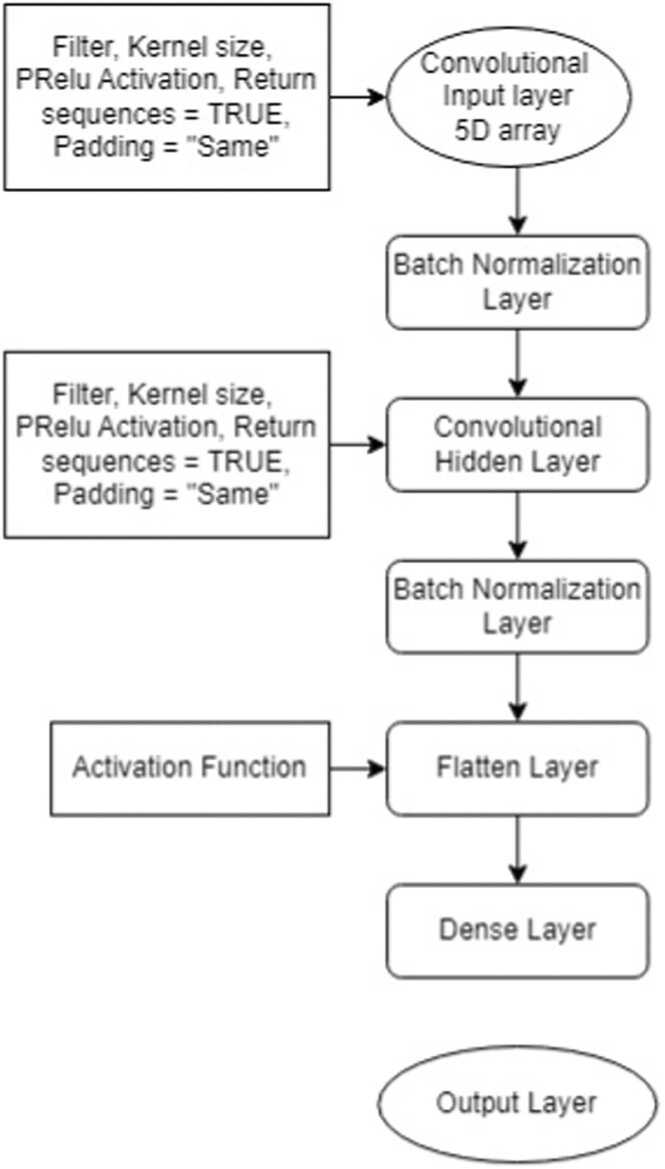
**:** ConvLSTM2D architecture for time-series genotype classification of plants.

For ConvLSTM2D, we created the 5-D input arrays. The ConvLSTM2D model’s system architecture (Fig. 4) was then used to construct a seven-layer network, which consists of the following layers: one input layer with the first batch normalization layer (instead of the first 2-D MaxPooling layer and dropout), one convolutional hidden layer with the second batch normalization layer (instead of the second 2-D MaxPooling layer and dropout), one flatten, one dense, and one output layer. The batch normalization layer shortened the learning time for the model and stabilized (regularized) the layer input arrays for a faster deep learning process [69]. The batch normalization layer was taken into consideration, which normalized an output data sequence from a prior connected layer (input or concealed layer). The flatten layer accepted the second batch normalization layer’s processed input sequence and wrapped it into a single 1-D vector. The bundled input sequence was extracted and interpreted by the dense layer that was directly connected to the model flatten layer before being sent on to an output layer. The argument *“return_sequences”* was used. If *“return_sequences”* is true then ConvLSTM layer returns a sequence as a tensor comprising of sequence, filters, rows, and columns. The ConvLSTM layer delivers only the most recent output with filters, rows, and columns if the input *“return_sequences”* is false. Finally, the classification with the highest score was chosen using the output layer with the *softmax* activation function. The best potential combination was selected based on network performance after experimenting with various ConvLSTM layers and filter counts. After considering multiple hyperparameter settings, the following model hyperparameters were considered for best accuracy: padding was set to *“Same”* and the input, hidden, and dense layers all used the *relu* activation function, but the model output layer used the *softmax* function having the exact number of neurons as genotypes in the dataset. The filter sizes for the input, hidden, and dense layers were set to 128, 256, and 1024 respectively. Our approach was to first pass each individual plant leaf area values through the feature extractor (CNN) to produce a fixed-length vector representation. This fixed-length vector embodies the features of each individual plant. The outputs of CNN for the sequence of plant area values were then passed onto a sequence learning module (LSTM). At this stage, the LSTM attempted to classify the plants via analyzing the sequences of the features that were extracted from time-series area growth of plants and by considering their temporal variations.

#### 2.7.7 k-fold cross-validation

We used k-fold (k = 10 in our analysis) cross-validation predictions as projected values for the training data. Here, we (randomly) divided the training data d into k equal-size folds or groups. A single fold is kept as the validation fold and used to test the algorithm once an algorithm has been trained on the other k-1 folds; then the process repeats for each of the folds. This way, each plant has a predicted genotype, obtained when the fold it belongs to is used as the validation fold. For this purpose, we used the “cross_val_score” method from sklearn library [82]. After performing k-fold cross-validation, we do not need any additional statistical tests to determine which model is better than which model because k-fold cross-validation is itself a statistical technique that provides a reliable estimate of the model’s performance. By repeating the training and testing process k times with different subsets of the data, we can obtain an estimate of the model’s generalization performance that is less affected by the noise in the dataset than a single train-test split.

#### 2.7.8 Hyperparameter optimization

The hyperparameter optimization is to define a tuple of hyperparameters that produces an optimal algorithm that minimizes the predefined loss function (i.e., cross-entropy loss function in our study) on a held-out validation set of the training data. In our study, we carried out hyperparameter optimization by first setting up a reasonable subset of the hyperparameter space of an ML algorithm, then evaluating it through cross-validation on the training data, during which an exhaustive grid search approach was used and the best was chosen as the “optimal”. For deep learning models, we similarly set up a reasonable subset of different groups of parameters and chose the parameters for which we acquired the best validation accuracy with the minimum validation loss. Besides, we set each deep learning model to 250 epochs. We employed the “EarlyStopping” approach [83] to stop iteration. Early stopping technique prevents overfitting and improves the generalization performance of the model. It involves monitoring the performance of the model on a validation set during the training process and stopping the training when the performance on the validation set starts to degrade. We used “patience = 15″. The “patience” parameter is a user-defined value that specifies the number of epochs to wait before stopping the training if the performance on the validation set does not improve. In our case, the patience is set to 15, the training process will continue for up to 15 epochs after the last time the validation loss improved. If the validation loss does not improve within the next 15 epochs, the training will stop since there was no observed decrease in validation loss after that certain epoch.

### 2.8 Evaluation

The effectiveness of the applied approaches in classifying the different genotypes is measured by four metrics: precision, recall, F1-score, and accuracy, which are commonly used to compare the performance of the traditional and deep learning algorithms. These metrics are defined as the following:

(3) *Recall* = *T P* / (*T P* + *FN*)

(4) *Precision* = *T P* / (*T P* + *FP*)

(5) *F*1 − *score* = 2 * Precision* *Recall* / (Precision + *Recall*)

(6) *Accuracy* = (*T P* + *TN*) / (*T P* + *TN* + *FP* + *FN*)

where TP, TN, FP and FN are the numbers of true positives, true negatives, false positives, and false negatives respectively. True positives are data points that have been marked as positive and are in fact positive. For example, model predicts a plant as “*wt”*, which truly belongs to *“wt”*. False positives are data points with a positive label but a negative one. In our study, if a plant of another genotype misclassified as *“wt”* genotype. True negatives are data points that are truly negative despite being classified as negative. False negatives are data items with negative labels but positive values.

## 3 Code availability

All codes implementing the models are available through GitHub (https://github.com/NazmusSakeef/Plant-Genomics-Using-Machine-Learning/tree/main/dataset).

## 4 Results and discussion

In our study, we analyzed 17 different photoreceptor deficient plant lines grown under three different twilight conditions. We first evaluated four traditional ML algorithms and ensemble stacking, then evaluated the performance of an additional six DL models. Here, we found that DL models consistently outperformed conventional machine learning models in terms of precision (P), recall (R), F1-Score (F1), and accuracy (Acc) (Table 1, Table 2, Table 3, Table 4, Table 5, Table 6). Using a total of 17 genotypes, we created different datasets with 4, 6, 9, and 17 genotypes “classes” (by merging some closely related genotypes), respectively, to assess each model’s performance, followed by the assessment of all algorithms on 3 separate datasets of 0-min, 30-min, and 90-min twilight growth conditions. We first evaluated traditional ML models for genotype classification in terms of precision, recall, F1-score, and accuracy (Table 1, Table 2, Table 3). Here, we found that for each of the four datasets with 4, 6, 9, and 17 genotype classes, SVM with polynomial kernel performed comparatively better than other traditional ML models (ANOVA *p-value*<0.05), achieving the highest precision, recall, F1-Score, and accuracy in all scenarios. Under the 0-min twilight condition, SVM achieved an average accuracy of 73%, 64%, 54%, and 40%, respectively, using 4, 6, 9, and 17 genotype classes, respectively. The maximum accuracy among all growth conditions was achieved by SVM for the 30-min twilight condition, where an average accuracy of 74%, 65%, 55%, and 41%, respectively, was obtained for 4, 6, 9, and 17 genotype classes. Observing that SVM with polynomial kernel performed better in classifying time-series area data of plants with higher dimensions, it seems that kernel selection and parameter search are particularly important in the performance of SVM. It has also been reported that the effectiveness of SVM generalization (estimation accuracy) required a good setting of the kernel parameters, hyper-parameters C, and gamma [84]. We found that SVM with polynomial kernel converged quickly, handled nonlinear problems, affected the complexity of model selection, had fewer numerical challenges, and non-linearly mapped samples into a higher dimensional space [85]. Next, our results indicated that Logistic Regression (LR) ranked the second-best for different twilight conditions. Here, we also found the optimal parameter setup of LR model by grid search method also plays an important role in achieving higher prediction.

**Table 1:** Classification results of ML models on 0-min twilight for different numbers of classes (genotypes).

Number of Classes	Model	P	R	F1	Acc
4 Classes	LR	0.75	0.73	0.73	0.72
	RF	0.68	0.67	0.67	0.68
	Boosting	0.71	0.68	0.68	0.70
	**SVC (poly)**	**0.73**	**0.73**	**0.72**	**0.73**
	Stacking	0.72	0.72	0.71	0.72
6 Classes	LR	0.63	0.62	0.62	0.62
	RF	0.61	0.59	0.59	0.60
	Boosting	0.60	0.59	0.60	0.60
	**SVC (poly)**	**0.65**	**0.63**	**0.64**	**0.64**
	Stacking	0.63	0.61	0.61	0.61
9 Classes	LR	0.49	0.50	0.49	0.50
	RF	0.52	0.51	0.51	0.51
	Boosting	0.53	0.52	0.52	0.52
	**SVC (poly)**	**0.57**	**0.54**	**0.54**	**0.54**
	Stacking	0.53	0.51	0.52	0.51
	LR	0.39	0.38	0.38	0.39
	RF	0.38	0.37	0.35	0.37
17 Classes	Boosting	0.39	0.39	0.38	0.39
	SVC (poly)	0.41	0.39	0.40	0.40
	Stacking	0.39	0.39	0.38	0.39

**Table 2:** Classification results of ML models on 30-min twilight for different numbers of classes (genotypes).

Number of Classes	Model	P	R	F1	Acc
4 Classes	LR	0.76	0.74	0.73	0.75
	RF	0.68	0.69	0.68	0.69
	Boosting	0.71	0.70	0.69	0.71
	**SVC (poly)**	**0.75**	**0.73**	**0.73**	**0.74**
	Stacking	0.73	0.73	0.72	0.73
6 Classes	LR	0.64	0.62	0.62	0.62
	RF	0.61	0.61	0.60	0.61
	Boosting	0.61	0.61	0.61	0.61
	**SVC (poly)**	**0.65**	**0.65**	**0.64**	**0.65**
	Stacking	0.64	0.62	0.62	0.62
9 Classes	LR	0.50	0.51	0.50	0.51
	RF	0.53	0.51	0.52	0.51
	Boosting	0.54	0.53	0.53	0.53
	**SVC (poly)**	**0.59**	**0.55**	**0.55**	**0.55**
	Stacking	0.54	0.52	0.52	0.52
17 Classes	LR	0.39	0.40	0.39	0.40
	RF	0.39	0.38	0.38	0.38
	Boosting	0.40	0.40	0.39	0.40
	**SVC (poly)**	**0.41**	**0.41**	**0.40**	**0.41**
	Stacking	0.40	0.40	0.39	0.40

**Table 3:** Classification results of ML models on 90-min twilight for different numbers of classes (genotypes).

Number of Classes	Model	P	R	F1	Acc
4 Classes	LR	0.74	0.74	0.74	0.74
	RF	0.68	0.68	0.68	0.68
	Boosting	0.69	0.68	0.68	0.69
	**SVC (poly)**	**0.73**	**0.73**	**0.73**	**0.73**
	Stacking	0.73	0.72	0.71	0.72
6 Classes	LR	0.63	0.62	0.62	0.62
	RF	0.61	0.59	0.59	0.60
	Boosting	0.60	0.59	0.60	0.60
	**SVC (poly)**	**0.65**	**0.63**	**0.64**	**0.64**
	Stacking	0.63	0.61	0.61	0.61
9 Classes	LR	0.49	0.50	0.49	0.50
	RF	0.52	0.51	0.51	0.51
	Boosting	0.53	0.52	0.52	0.52
	**SVC (poly)**	**0.57**	**0.54**	**0.54**	**0.54**
	Stacking	0.53	0.51	0.52	0.51
17 Classes	LR	0.39	0.38	0.38	0.39
	RF	0.38	0.37	0.35	0.37
	Boosting	0.39	0.39	0.38	0.39
	**SVC (poly)**	**0.41**	**0.39**	**0.40**	**0.40**
	Stacking	0.39	0.39	0.38	0.39

**Table 4:** Classification results of Deep learning (DL) models on 0-min twilight for different numbers of classes (genotypes).

Number of Classes	Model	P	R	F1	Acc
4 Classes	FCN	0.74	0.74	0.73	0.74
	Conv2D	0.77	0.75	0.76	0.76
	**ConvLSTM2D**	**0.79**	**0.80**	**0.79**	**0.79**
	Encoder	0.76	0.74	0.75	0.74
	LSTM	0.73	0.73	0.72	0.72
	ResNet	0.73	0.74	0.72	0.74
6 Classes	FCN	0.68	0.63	0.62	0.63
	Conv2D	0.65	0.65	0.64	0.65
	**ConvLSTM2D**	**0.66**	**0.66**	**0.66**	**0.66**
	Encoder	0.63	0.61	0.62	0.63
	LSTM	0.63	0.63	0.62	0.63
	ResNet	0.64	0.65	0.64	0.65
9 Classes	FCN	0.53	0.50	0.51	0.51
	Conv2D	0.53	0.50	0.51	0.51
	**ConvLSTM2D**	**0.57**	**0.55**	**0.56**	**0.56**
	Encoder	0.54	0.54	0.53	0.53
	LSTM	0.49	0.48	0.48	0.48
	ResNet	0.52	0.49	0.50	0.50
17 Classes	FCN	0.39	0.38	0.39	0.39
	Conv2D	0.43	0.41	0.42	0.42
	**ConvLSTM2D**	**0.45**	**0.44**	**0.44**	**0.44**
	Encoder	0.44	0.43	0.43	0.43
	LSTM	0.41	0.40	0.40	0.40
	ResNet	0.41	0.41	0.41	0.41

**Table 5:** Classification results of Deep Learning (DL) models on 30-min twilight for different numbers of classes (genotypes).

Number of Classes	Model	P	R	F1	Acc
4 Classes	FCN	0.74	0.74	0.74	0.74
	**Conv2D**	**0.78**	**0.78**	**0.78**	**0.78**
	ConvLSTM2D	0.82	0.80	0.80	0.81
	Encoder	0.77	0.76	0.77	0.76
	LSTM	0.75	0.74	0.74	0.74
	ResNet	0.75	0.75	0.74	0.75
6 Classes	FCN	0.69	0.64	0.63	0.64
	**Conv2D**	**0.66**	**0.67**	**0.67**	**0.67**
	ConvLSTM2D	0.70	0.68	0.69	0.69
	Encoder	0.66	0.66	0.66	0.66
	LSTM	0.65	0.65	0.64	0.65
	ResNet	0.67	0.66	0.65	0.66
9 Classes	FCN	0.55	0.52	0.53	0.53
	Conv2D	0.56	0.53	0.54	0.54
	**ConvLSTM2D**	**0.59**	**0.56**	**0.57**	**0.57**
	Encoder	0.56	0.56	0.56	0.56
	LSTM	0.51	0.50	0.51	0.51
	ResNet	0.54	0.51	0.52	0.52
17 Classes	FCN	0.43	0.41	0.42	0.42
	Conv2D	0.44	0.42	0.43	0.43
	**ConvLSTM2D**	**0.46**	**0.45**	**0.45**	**0.45**
	Encoder	0.45	0.44	0.44	0.44
	LSTM	0.42	0.42	0.42	0.42
	ResNet	0.44	0.43	0.44	0.44

**Table 6:** Classification results of Deep learning (DL) models on 90-min twilight for different numbers of classes (genotypes).

Number of Classes	Model	P	R	F1	Acc
4 Classes	FCN	0.74	0.74	0.73	0.74
	Conv2D	0.77	0.76	0.77	0.77
	**ConvLSTM2D**	**0.80**	**0.79**	**0.80**	**0.80**
	Encoder	0.76	0.76	0.75	0.75
	LSTM	0.74	0.73	0.74	0.73
	ResNet	0.73	0.73	0.72	0.73
6 Classes	FCN	0.66	0.64	0.64	0.64
	Conv2D	0.65	0.65	0.64	0.65
	ConvLSTM2D	0.68	0.67	0.68	0.67
	Encoder	0.64	0.63	0.64	0.63
	LSTM	0.62	0.61	0.62	0.62
	ResNet	0.65	0.64	0.64	0.64
9 Classes	FCN	0.53	0.50	0.51	0.50
	Conv2D	0.53	0.52	0.52	0.52
	ConvLSTM2D	0.57	0.55	0.56	0.56
	Encoder	0.54	0.54	0.53	0.53
	LSTM	0.49	0.49	0.49	0.49
	ResNet	0.52	0.50	0.50	0.51
17 classes	FCN	0.40	0.39	0.40	0.40
	Conv2D	0.43	0.41	0.42	0.42
	ConvLSTM2D	0.45	0.44	0.44	0.44
	Encoder	0.44	0.43	0.43	0.43
	LSTM	0.42	0.42	0.41	0.41
	ResNet	0.41	0.40	0.41	0.41

We also found that stacking also achieved comparable results to SVM and LR. However, given its lower complexity (i.e., it’s easier to define, train, and maintain), the base model should be used instead of the stacking ensemble if it performs as well or better [86]. This suggests that the combination of multiple ML models can be more robust. Lastly, Random Forest (RF) performed the least well for almost all analyses. The RF classifier is known for being more susceptible to dataset noise than the other methods. Further, it has demonstrated more robust performance with larger datasets relative to that used here [87]. RF models have also been reported to produce strong results for multi-class classification problems; however, here we find them falling short of SVM for our time-series dataset.

For the DL models, we found that for each of the class sizes (4, 6, 9, and 17, respectively), ConvLSTM2D performed comparatively better all the other models (Tables 4–6), by achieving the highest precision (P), recall (R), F1-Score (F1), and accuracy (Acc) in all scenarios (ANOVA *p-value*<0.05). Encoder model performed almost as well as ConvLSTM2D model, while LSTM model performed comparatively poorer than the other DL models. In more detail, under the 0-min twilight light condition, ConvLSTM2D achieved an average accuracy of 79%, 66%, 56%, and 44% accuracy, respectively, for class sizes of 4, 6, 9, and 17. The maximum accuracy was reached by ConvLSTM2D under the 30-min twilight condition, with an average accuracy of 81%, 69%, 57%, and 45% for class sizes of 4, 6, 9, and 17, respectively.

For the ConvLSTM2D model, we incorporated the features of both the CNN and LSTM models together. Here, the most descriptive features in the data are automatically found and extracted by CNNs, while LSTM manages the dynamic behaviors occurring throughout plant growth and development. LSTM attempts to classify the plants by analyzing the sequence of features that are extracted from the daily time-series of plant growth and by considering their temporal variations, which in combination, provide better classification results than other models. Collectively, our findings indicate that ConvLSTM2D is ideal for time-series across multiple scales, genotypes and growth conditions, which is likely due to the ability of ConvLSTM2D to incorporate multidimensional datasets such as ours.

**Fig. 5:**
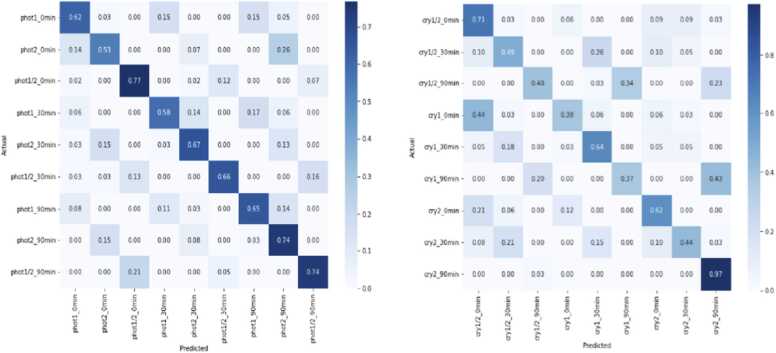
Performance of ConvLSTM2D model on the phototropin group (left) and the cryptochrome group (right) for different dawn and dusk periods (0, 30, and 90-minutes).

To assess the performance of the ConvLSTM2D modelsat the intra-group level, we examined its ability to classify different genotypes of the phytochromes group (*phyA* – *phyE*; class size = 5), the phototropins group (*phot1*, *phot2*, & *phot1/2*; class size = 3) and the cryptochromes group (*cry1*, *cry2* & *cry1/2*; class size = 3). We evaluated its performance by presenting the confusion matrix (Fig. 5). We selected 3 classes from 3 different light conditions to make a total of nine potential ‘classes’ for each group. From this analysis, we extracted precision, recall, F1-score, and accuracy of 67%, 66%, 66%, and 66%, respectively for the phototropin group, while achieving precision, recall, F1-score, and accuracy of 57%, 56%, 56%, and 56% respectively for the cryptochrome group (Table 7). These suggest that ConvLSTM2D successfully classified different genotypes under different twilight conditions using multi-scale time-series data, and based on the confusion matrix, we can readily identify which genotypes are classified/misclassified to which class (Fig. 5).

**Table 7:** Performance of the ConvLSTM2D model on different genotype groups grown under different twilights (0, 30, and 90-minutes).

Group	Time	P	R	F1	Accuracy
Phytochrome	0 min	0.61	0.61	0.61	0.61
	30 min	0.66	0.66	0.66	0.66
	90 min	0.61	0.61	0.61	0.61
Phototropins	0 min	0.72	0.72	0.72	0.72
	30 min	0.74	0.73	0.73	0.73
	90 min	0.71	0.70	0.71	0.71
ztl/fkf1/lkp2	0 min	0.66	0.66	0.66	0.66
	30 min	0.66	0.66	0.66	0.66
	90 min	0.64	0.65	0.64	0.65
Cryptochrome	0 min	0.66	0.66	0.66	0.66
	30 min	0.67	0.66	0.67	0.67
	90 min	0.64	0.64	0.64	0.64

The ConvLSTM2D model performance analysis data for each group of related photoreceptor deficient plants is shown in Table 4, Table 5, Table 6, from which we observed several similarities between the “classes” inside each group. For instance, we see a more similar intra-/inter-day growth pattern between plants of genotypes *phot2* and *phot1/2*, relative to *phot1* inside the phototropin group, with *phot2* plants consistently miscategorized as *phot1/2*. Using a confusion matrix, we then found that under a 0-min and 30-min we find *phot2* plants misclassified as *phot1/2* plants 12% (0.12) and 31% (0.31) of the time, respectively, while under a 0-min twilight, *phot1/2* plants are misclassified as *phot2* plants 24% (0.24) of the time (Supplemental Fig. 1).

**Fig. 6:**
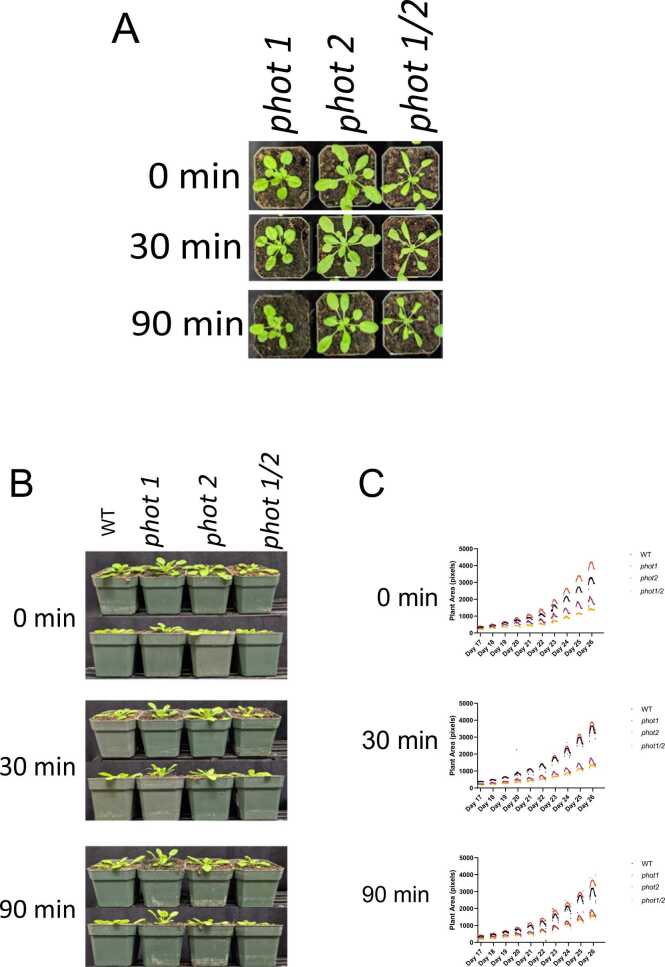
Phototropin mutants details. (A) Pictures from the top of phototropin mutants *phot1*, *phot2*, and *phot1/2* under different light treatments. (B) Pictures from the side of *phot1*, *phot2* and *phot1/2* under different light treatment.(C) Plant area in pixels of each phototropin mutant over 10 days calculated with PlantCV.

Phototropins are particularly interesting as they control leaf movement and positioning in response to blue light [87]. Compared to WT plants, *phot1* and *phot2* plants possess phototropic bending towards high and low fluence blue light, respectively, while *phot1/2* plants have no phototropic responses to either low or high blue light. By collecting time-series data over multiple timescales, our surface area data reveals specific intra-day grow patterns for each genotype (Fig. 6). In most genotypes, area data is not linear over the course of a day, but rather forms a peak at mid-day. This suggests that changes in surface area may result from leaf movement across the day. Correspondingly, we hypothesize that this creates a signature feature that the ConvLSTM2D model can be successfully used to distinguish unique genotypes. In the case of the phototropins, we can see that *phot2* plant area is similar to that of *phot1/2*, with both being relatively “flat” across the day in a twilight-dependent manner. This is especially apparent in the 90 min twilight condition, where *phot2* and *phot1/2* possess minimal variation, which correlates with their mis-categorization, suggesting that changes in leaf area, mediated by leaf movement, create a unique feature over time that DL models can utilize to detect differences in plant genotypes. In particular, the ConvLSTM2D model using time-series data across multiple scales represents the best model for differentiating genotypes grown under different light conditions.

We note that, for LSTM, the model architecture has a memory cell that maintains a current state over various sequential instances and non-linear dependencies that regulate information entering and exiting the cell. Bi-LSTM (an enhanced architecture of LSTM) models are typically more effective when managing contextual information since their output at a given time depends on both the prior and subsequent segments. To comprehend past and future information, the Bi-LSTM architecture contains two layers: forward and backward directions. Every series of events submitted to an LSTM is processed one time step at a time. A vector holding data about the current and prior time steps is passed from one time step to the next until it reaches the last one. However, the informational content of the vector will eventually be constrained by its fixed size [41]. The information from the previous inputs runs the danger of being lost or diluted, especially for longer sequences. On the other hand, CNN models such as Conv2D, can automatically identify patterns and extract features from the input data, applying a series of convolution layers in successions such as weight-sharing filters and dimension-reducing pooling layers. These features are then passed to a series of dense layers for classification (or regression). Two-dimensional CNN (Conv2D) are commonly used in computer vision applications to interpret time as a spatial dimension.

## 5 Conclusion

In this study, we demonstrate the potential of machine learning (ML) and deep learning (DL) methods on multi-scale time-series data for classifying *Arabidopsis thaliana* plants genotypes grown under different light conditions. In doing this, we developed a dataset of 17 different genotypes subjected to three different twilight growth conditions. Each of the tested ML and DL algorithms exhibits a capacity for capturing subtle growth features which would otherwise escape manual inspection. We found that DL algorithms perform comparatively better, likely owing to their interpretation of time-series data. Among the DL algorithms we tested, our results find that ConvLSTM2D performed comparatively better than other DL algorithms in all measurements including precision, recall, F1-score, and accuracy, using the leaf area values extracted from the plant growth images for genotype classification/prediction. Our findings successfully integrated time-series growth data across scales (intra- and inter-day), genotypes, and growth conditions, establishing a new baseline from which more variables can be integrated in order to assess more complex plant traits. However, there are several areas for future work. First, our study focused on a single plant species, and so it would be valuable to expand our analysis to include a broader range of plant species. Second, our study utilized plant area data, and we hypothesize that more plant growth characteristics such as leaf greenness may further improve genotype classification by ML models. Collectively, in undertaking this study have expanded our ability to make genotype-phenotype connections in the plant sciences using machine learning and deep learning algorithms.

## Funding

This work was supported by the Natural Sciences and Engineering Research Council (NSERC) of Canada and Alberta Innovates.

## CRediT authorship contribution statement

NS performed all ML and DL analyses with assistance from JC and CK. SS and CL performed all plant growth and image acquisition. CK and SS performed all image analysis and data extraction for use in ML and DL. GL and RGU conceived of the study. The manuscript was written and edited by NS, GL, SS and RGU.

## Declaration of Competing Interest

The authors declare no conflicts of interest.
