# Supplementary material for: Machine learning classification of plant genotypes grown under different light conditions through the integration of multi-scale time-series data
Source: Comput Struct Biotechnol J. 2023 May 23;21:3183–95. doi: 10.1016/j.csbj.2023.05.005 (PMC10275741; doi:10.1016/j.csbj.2023.05.005)
Supplement: Supplementary file 1 — Supplementary material. [file mmc1.pdf]

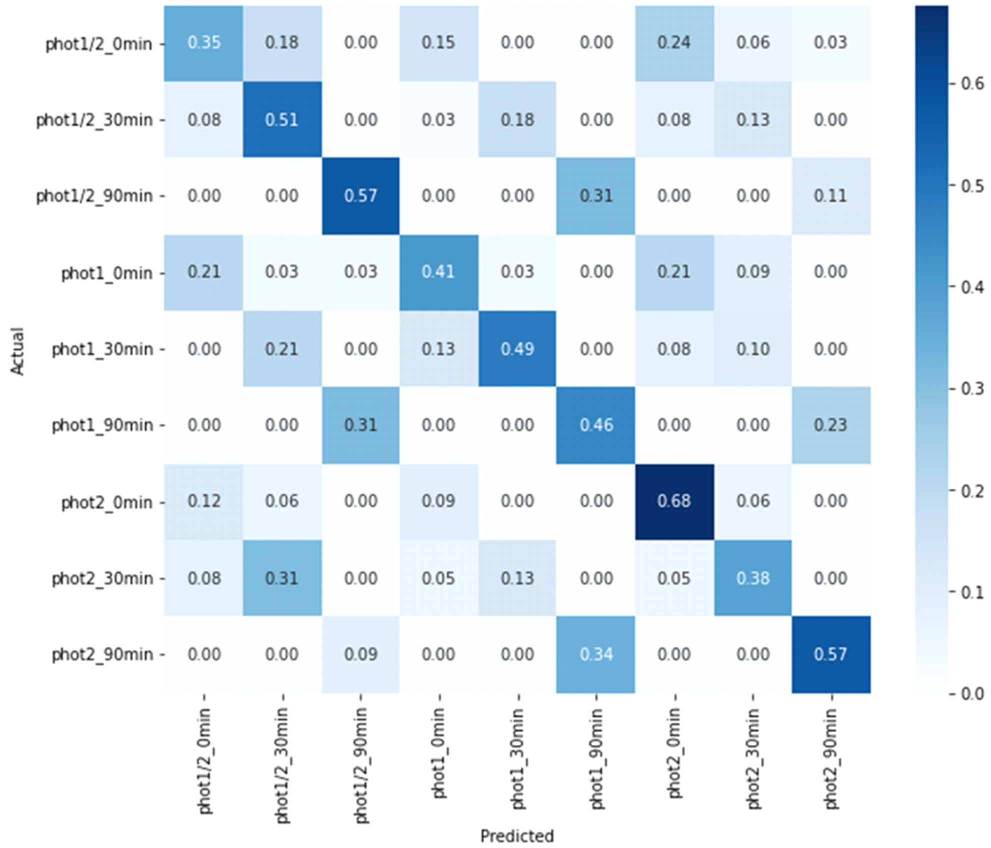

Supplemental Figure 1: Performance of ConvLSTM2D model on the Phototropin mutant plants grown under different twilights (0, 30, and 90-minutes). The confusion matrix demonstrates that phot2 plants are misclassified into phot1/2 plants. For example, phot2_0min has a misclassification of 0.12 into phot1/2_0min, which means the model mistakenly predicted 12% of phot2 plants as phot1/2 plants (False Negative). Again, phot2_30min plants get misclassified as phot1/2_30min plants with a misclassification rate of 0.31 and phot1/2_0min plants have a false negative rate of 0.24 with phot2_0min plants.
